# Type IV Sacrococcygeal Teratoma Associated with Urogenital Sinus: Difficulties in the Prenatal Differential Diagnosis

**Published:** 2013-01-01

**Authors:** Zeki Sahinoglu, Aysenur Cerrah Celayir, Mehmet Resit Asoglu, Nahit Özcan

**Affiliations:** 1 Department of Obstetrics and Gynecology, Zeynep Kamil Women and Child Diseases Education and Research Hospital Istanbul, Turkey.; 2 Department of Pediatric Surgery, Zeynep Kamil Women and Child Diseases Education and Research Hospital Istanbul, Turkey.; 3 Sonomed Medical Imaging Center, Istanbul, Turkey.

**Keywords:** Sacrococcygeal teratoma, Cloacal anomaly, Urogenital sinus, Fetal ultrasonography, Fetal MRI

## Abstract

Sacrococcygeal teratoma (SCT) is being more often detected due to availability of prenatal ultrasonography. Type IV SCT could be misdiagnosed as cloacal abnormalities due to the pelvic midline cystic mass associated with renal malformations and obstructive uropathy during the pregnancy. We discuss difficulties in the prenatal differential diagnosis of SCT and urogenital sinus in a 26-year-old pregnant woman, admitted to our prenatal diagnosis centre for a detailed US for a pre-sacral mass.

## INTRODUCTION

Sacrococcygeal teratoma (SCT) is the most common congenital tumor in the newborn period, occurring in 1 in 40,000 infants with a marked female predominance (75%) [1]. These tumors are seen as heterogeneous well-circumscribed predominantly external caudal masses. Sacrococcygeal teratomas are classified into four types according to Altman Classification [2]. Type IV SCTs are entirely internal and cystic without external component. On prenatal ultrasonography, type IV SCT may mimic cloacal anomalies, because both of them could be seen as pelvic midline cystic mass. Association of the cloacal abnormalities and sacrococcygeal teratomas are very rare. Anorectal malformation, sacral bony abnormality and presacral mass were first described as Currarino triad by Bryant in 1838 [3]. Herein, we present difficulties in prenatal ultrasonographic diagnosis of intrapelvic cystic mass; this was resolved by fetal magnetic resonance imaging (MRI).

## CASE REPORT

A 26-year-old pregnant women, gravida 1, para 0, was admitted to our prenatal diagnosis centre for detailed ultrasonography examination. Obstetric history was unremarkable. A single fetus was detected at 26 gestational weeks. A presacral located cystic mass measuring 45 × 55 × 40 mm was seen on ultrasound examination (Fig. 1). On doppler ultrasound, the cystic structure with both umbilical arteries around was considered an enlarged distally obstructed fetal bladder initially (Fig. 2). However, the bladder was later identified more superiorly. The amniotic fluid index was 129mm. Diagnosis of the cystic mass was unclear and a fetal MRI was performed, which delineated a large cystic mass with minimal internal echogenicity area, the type IV teratoma (big arrow head). The white arrow demonstrates a cystic mass considered as a hydrometrocolposis (Fig. 3).

**Figure:**
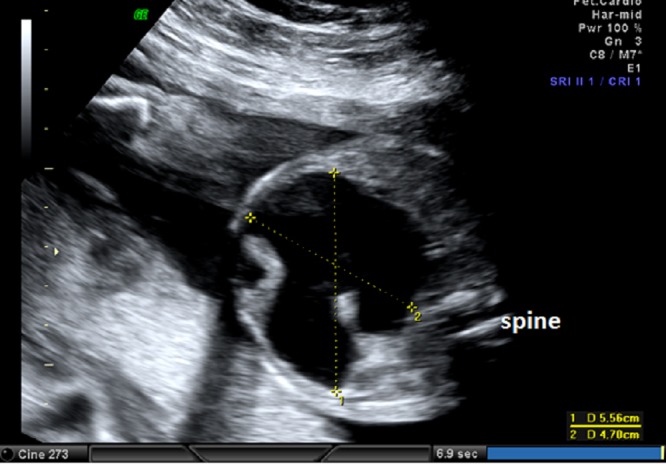
Figure 1: The pelvic cystic structure (55 x 47 mm) with irregular borders.

**Figure:**
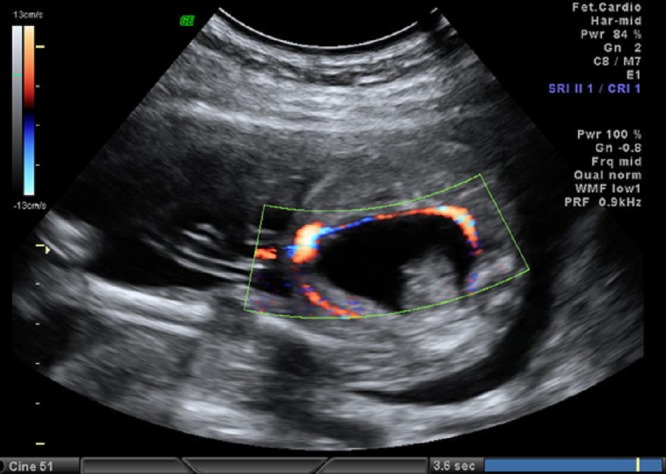
Figure 2: The cystic structure with both umbilical arteries around.

**Figure:**
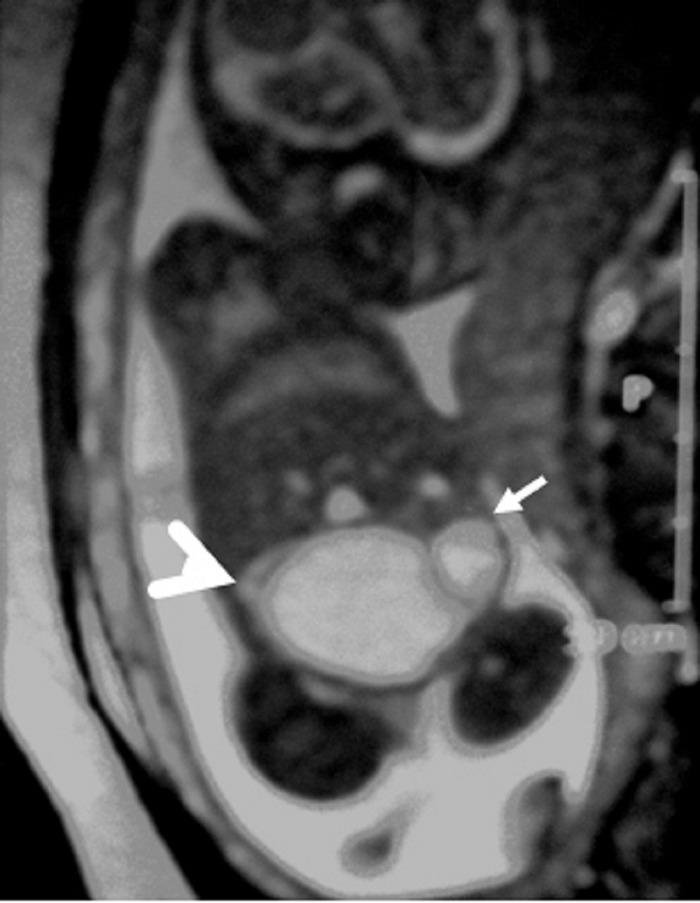
Figure 3: Fetal MRI showing type IV teratoma (big arrow head) and the white arrow demonstrates hydrometrocolpos.

In the axial plane, besides type IV SCT (big arrow head) and hydrometrocolpos (white arrow); both kidneys (white asterix) appeared in normal loca¬tion and anatomy (Fig. 4). In the coronal plane fetal bladder (black empty arrow) was noticed (Fig. 5).

**Figure:**
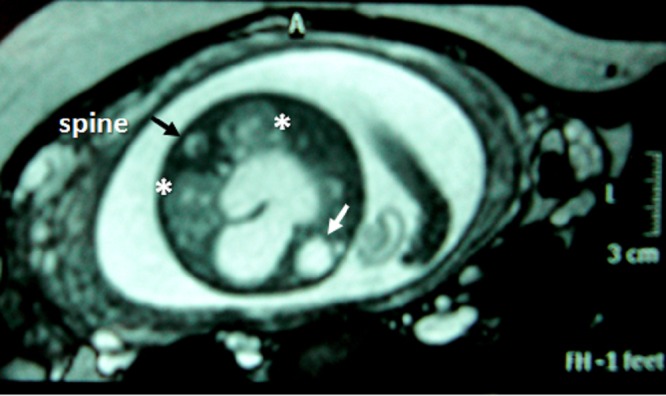
Figure 4: Fetal MRI axial plane.

**Figure:**
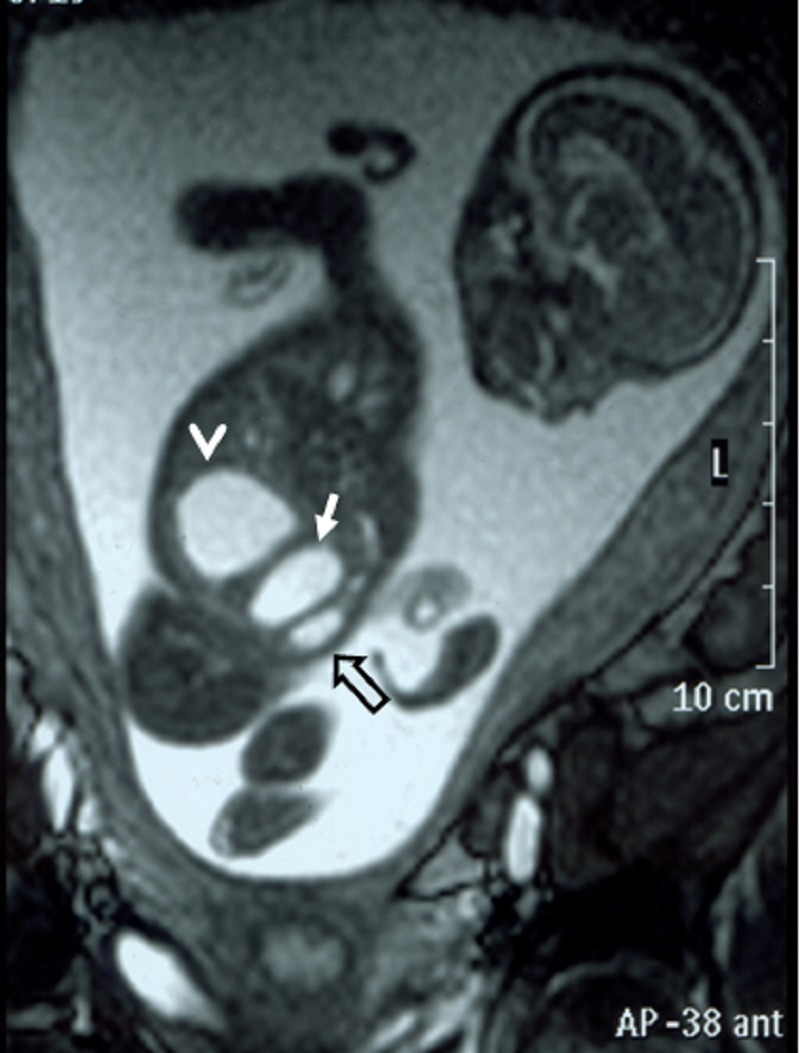
Figure 5: Fetal MRI coronal plane.

A 3350 g baby was delivered at 37 weeks by caesarian section. Diagnosis of type IV SCT was confirmed postnatally, but she also had hydrometrocolpos due to urogenital sinus. At the postnatal ultrasonographic examination, hydrometrocolpos and bilateral hydroureteronephrosis were confirmed. SCT was excised totally along with coccygectomy, and an abdominal vaginostomy was created due to hydrometrocolpos. Follow up was uneventful; she is waiting for definitive operation for urogenital sinus.

## DISCUSSION

The differential diagnoses of intrapelvic cystic masses include meconium pseudocyst, enteric duplication cyst, fetal ovarian cyst, hydrocolpos and its causes, dilated bowel, bowel duplication, mesenteric cyst, rectal duplication, cystic neuroblastoma, teratoma, or bladder duplication [5, 6]. A detailed fetal ultrasonographic scan and fetal MRI are the main diagnostic tools. Because of acoustic shadowing by the fetal pelvic bones, sonography cannot always define the tumour extent [7]. MRI provides better anatomic resolution, regardless of fetal position. However, there are no large studies published comparing the efficiencies of fetal ultrasound and MRI in SCT cases [1, 7, 8]. In a study comparing the diagnostic advantages of prenatal ultrasound and MRI in SCT, it was reported that the cephalad extension detected in fetal MRI was misdiagnosed in 36% of fetuses by ultrasound; however, the authors mentioned that trans-abdominal Doppler sonography was found sufficient for assessing the extension of cystic and extra-pelvic SCT [9]. 

The intrapelvic extent of the mass is variable and some teratomas are purely intrapelvic, as in our case. A few differential diagnoses may be discussed when a pelvic midline cystic mass is discovered in a female fetus. In addition, observation of hydrometrocolpos illustrates the diagnostic problems generated by the detection of a pelvic midline cystic mass in a female fetus. A precise analysis of the structure of the mass and its relationships with the pelvic organs and the spine may lead to the diagnosis [4,5,8-10]. In favor of SCT were the location of the mass (very posterior, before the spine), the posteroinferior part of the mass pointing towards the coccyx, the inferior heterogeneous part of the mass possibly corresponding to a tissue component and the presence of a uterus with a normal signal. The second differential diagnosis was a cloacal anomaly because of the absence of visibility of the T1 hypersignal of meconium in the rectum. The absence of visibility of the T1 hypersignal of the meconium in the rectum is traditionally supposed to be highly suggestive of a cloacal anomaly, but may also be explained by the emptiness of the rectum, compressed by the mass [10, 11]. The third differential diagnosis could be anterior meningocele [11]. This midline pelvic cystic mass communicates with the sacral canal and is associated with bone abnormalities of the sacrum (hemihypoplasia or scimitar configuration) or the dorsal spine; dysraphism, gastrointestinal (anorectal malformation) or genitourinary abnormalities may also be present. Presacral meningocele may be isolated or associated with neurofibromatosis type I or Currarino triad. 

It is quite difficult to diagnose accurately urogenital or cloacal malformations by ultrasound in the fetus. The common channel in urogenital sinus, that could be sometimes atretic or obstructive, gives rise to vaginal, uterine and vesical distension. This observation illustrates the diagnostic difficulties in the presence of a pelvic midline cystic mass in a fetus. A detailed consideration of the structure of the mass and its relationships with the other pelvic organs and spine seems to be crucial in the precise diagnosis in combine anomalies. 

In conclusion, accurate prenatal diagnosis has a crucial role in the perinatal management and planning surgical options in pelvic cystic masses. The presence of unusual pelvic masses in fetus can add to the complexity of abnormality; however, most of these lesions can be detected by careful examination with US, Doppler, and MRI. Additional imaging techniques seem to be facilitating factor in counseling the parents, as well as promoting the perception of the fetal malformations by the family.

## Footnotes

**Source of Support:** Nil

**Conflict of Interest:** None
